# Lipidomics dataset of PTEN deletion-induced optic nerve regeneration mouse model

**DOI:** 10.1016/j.dib.2020.106699

**Published:** 2020-12-26

**Authors:** Jennifer Arcuri, Shane Hegarty, Zhigang He, Sanjoy K. Bhattacharya

**Affiliations:** a Bascom Palmer Eye Institute, Miller School of Medicine at University of Miami, Miami, FL, 33136, USA; b Miami Integrative Metabolomics Research Center, Miami, FL, 33136, USA; c Molecular Cellular Pharmacology Graduate Program, University of Miami, Miami, FL, 33136, USA; d F.M. Kirby Neurobiology Center, Department of Neurology, Boston Children's Hospital, Harvard Medical School, 300 Longwood Avenue, Boston, MA 02115, USA

**Keywords:** Regeneration, CNS Injury, Optic Nerve, PTEN, Lipids

## Abstract

The optic nerve is part of the mammalian adult central nervous system (CNS) and has limited capability to regenerate after injury. Deletion of phosphatase and tensin homolog (PTEN), a negative regulator of the PI3 kinase/Akt pathway, has been shown to promote regeneration in retinal ganglion cells (RGCs) after optic nerve injury [1]. We present the lipidome of adult PTEN^loxP/loxP^ mice subjected to intravitreal injection of adeno-associated viruses expressing Cre (AAV-Cre) as a model of CNS neuroregeneration. At 4 weeks old, PTEN^loxP/loxP^ mice were intravitreally-injected with 2–3 μl of either AAV-Cre (KO) or AAV-PLAP (control), and two weeks later optic nerve crush was performed. At indicated time-points after crush (0 days, 7 days, 14 days), mice were euthanized and optic nerves were immediately dissected out, and then flash frozen on dry ice. A modified Bligh and Dyer [2] method was used for lipid extraction from the optic nerves, followed by liquid chromatography-mass spectrometry (LC MS-MS) lipid profiling using a Q-Exactive Orbitrap instrument coupled with Accela 600 HPLC. The raw scans were analysed with LipidSearch 4.2 and the statistical analysis was conducted through Metaboanalyst 4.0. This data is available at Metabolomics Workbench, study ID ST001477.

**Specifications Table**

**Table:** 

Subject	Ophthalmology
Specific subject area	Lipids of the regenerating optic nerve
Type of data	Table Image Chart Graph Figure Chromatograms Spectra
How data were acquired	Liquid Chromatography Q-Exactive Orbitrap Mass Spectrometry
Data format	Raw Analyzed Filtered
Parameters for data collection	Intravitreal Injections of AAV-Cre (KO) or AAV-PLAP (control) and optic nerve crush on 4-week-old Pten^loxP/loxP^ mice
Description of data collection	Optic nerves of Pten^loxP/loxP^ (KO and control) mice were collected at 0, 7 and 14 days post optic nerve crush. Lipids were extracted and analysed by untargeted LC-MS/MS. These data also relates to previous optic nerve regeneration after crush induced by Zymosan+CPT-cAMP [3], Wnt3a [4] and optogenetic stimulation [5]. A supplementary table of the data is available with this manuscript.
Data source location	Bascom Palmer Eye Institute, Miller School of Medicine at University of Miami, Miami, FL 33136, USA
Data accessibility	Study ID ST001477at Metabolomics Workbench Repository: https://www.metabolomicsworkbench.org/data/DRCCMetadata.php?Mode=Project&ProjectID=PR001001 MetaboAnalyst 4.0 (https://www.metaboanalyst.ca) web based free tool can be used to analyse these data.

## Value of the Data

• The data provided pertain to the lipid changes that occur in the optic nerves of Pten^loxP/loxP^ (KO and control) mice after optic nerve crush injury and subsequent axon regeneration.
• The data is useful for the future studies of central nervous system (CNS) nerve injury and regeneration promoted using different genetic and pharmacological treatments.
• The data can be employed for the development of databases for future targeted lipidomics experiments for CNS neuronal regeneration.

## 1 Data Description

**Fig. 1:**
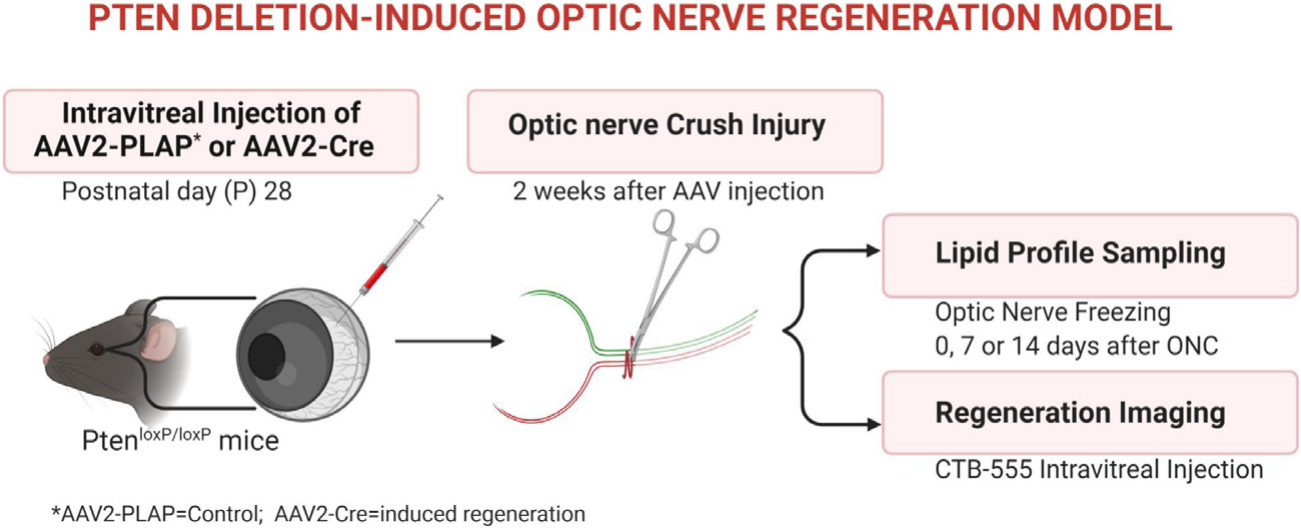
Schematic representation of PTEN deletion-induced optic nerve regeneration model. The dataset consists of the following experimental groups: PTEN 0, 7 and 14 days after optic nerve crush (P0, P07, P14) or Control (C0, C07, C14).

**Fig. 2:**
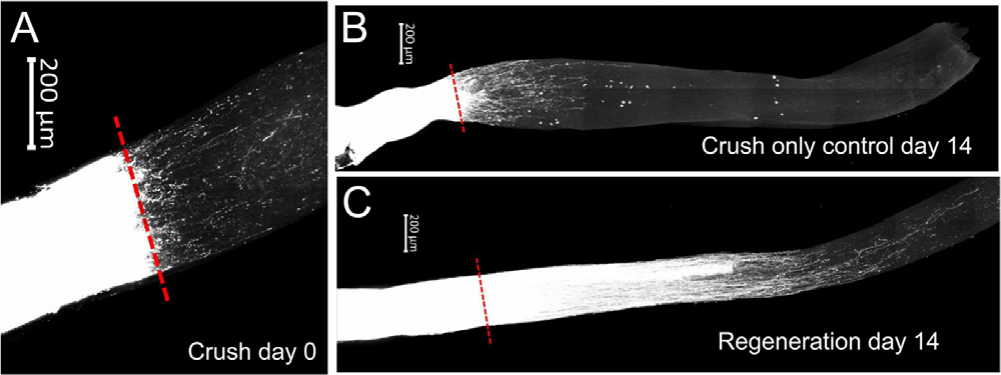
Schematic representation of PTEN deletion-induced optic nerve regeneration model. The schematic images consists of the following experimental groups: (A) PTEN-AAV2-cre 0 day (B) Control AAV2-PLAP 14 days and (C) PTEN AAV2-cre 14 days after optic nerve crush as indicated. Red line indicated crush site.

**Fig. 3:**
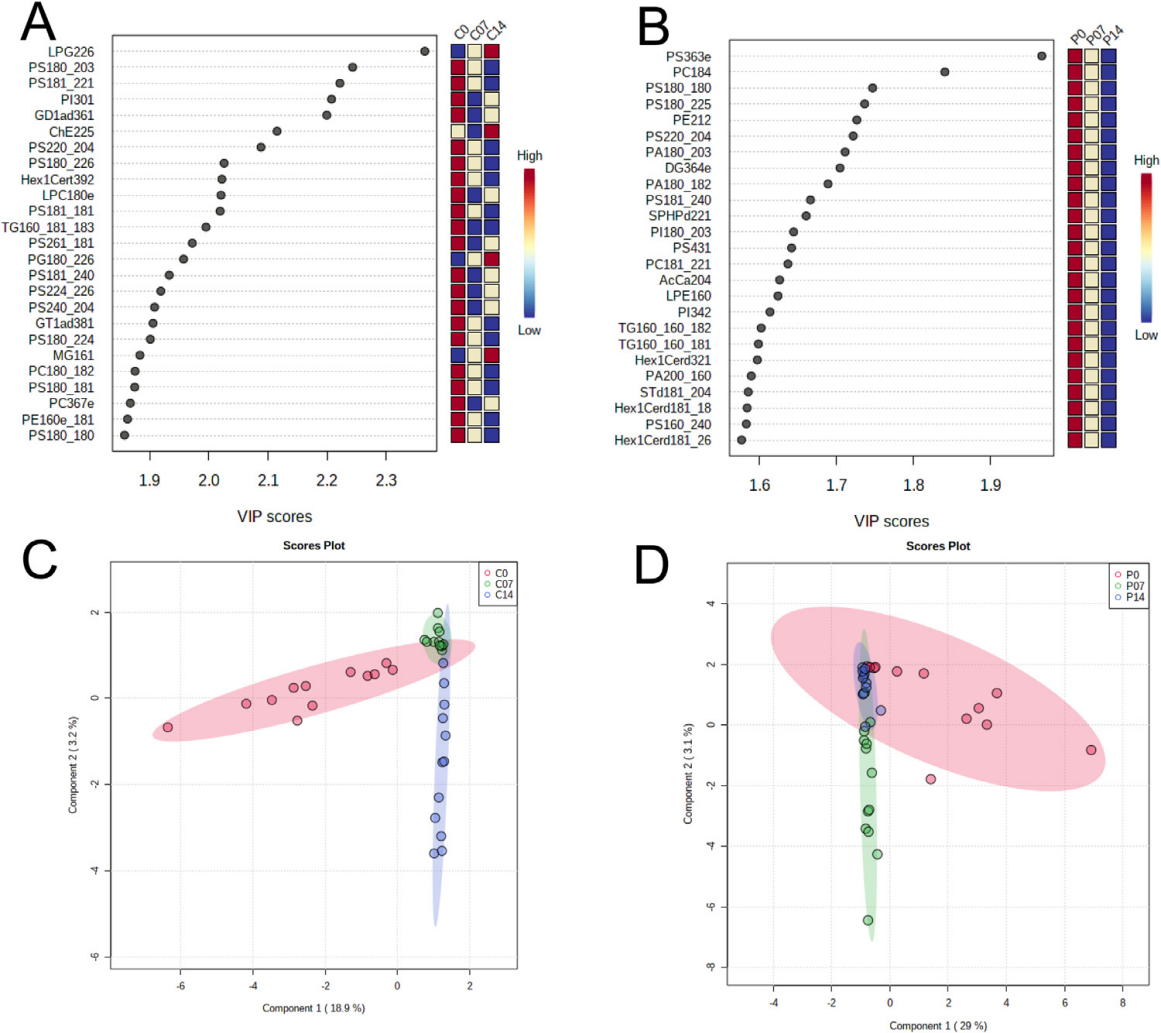
The statistical features of lipid profiles. Important features identified by partial least square-discriminant analysis (PLS-DA) and variable importance in projection (VIP) scores have been presented (A-B). The colored boxes on the right indicate the relative concentrations of the corresponding metabolite in controls (A) and KO (B).Scores plot between the selected principal components (C-D). The variances are shown in parentheses.

**Fig. 4:**
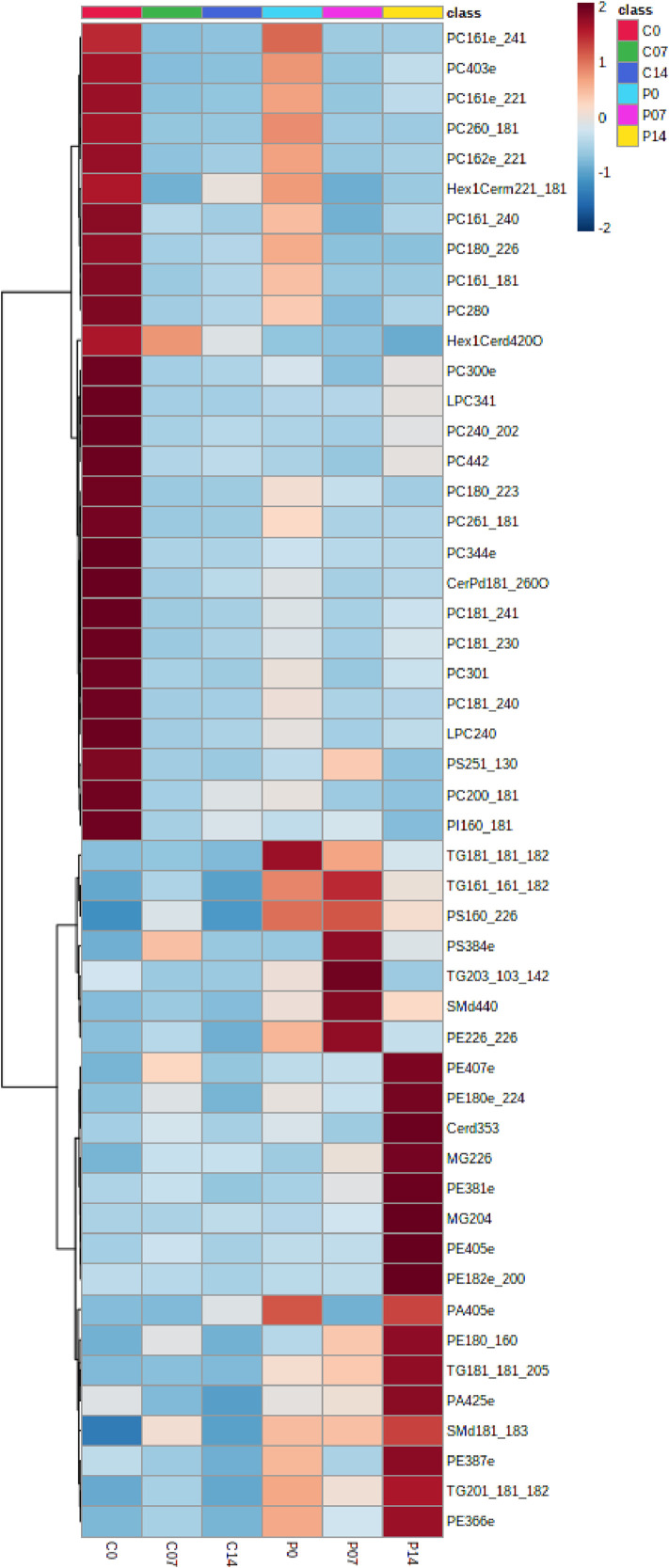
Heatmap of the lipid abundances. Changes post optic nerve crush of control (C0, C07, C14) and PTEN KO (P0, P07, P14). Control and PTEN KO refers to AAV2-PLAP and AAV2-Cre respectively. (Distance measure using euclidean, and clustering algorithm using ward.D)

Lipid profiling was performed on PTEN deletion-induced optic nerve regeneration mouse model and controls. At postnatal day 28, PTEN^loxP/loxP^ mice were subjected to intravitreal injection of adeno-associated viruses expressing Cre (AAV2-Cre) or control placenta alkaline phosphatase (AAV2-PLAP). Optic nerve crush (ONC) was performed 2 weeks after the AAV injections and at three different time points (0, 7, 14 days post crush) the nerves were collected for lipidomics (Fig. 1). Cholera toxin subunit B conjugated with Alexfluor555 (CTB-555) was used for anterograde labelling of the RGCs on crush day and 14 days post AVV injections. The images were taken with a Zeiss LSM 710 multiphoton confocal microscope (Fig. 2). Lipids were extracted using a modified Bligh and Dyer [2] method with an EquiSplash standard and analyzed using reversed-phase high-performance liquid chromatography (HPLC) coupled to a Q Exactive mass spectrometer. Lipid identification and relative quantification were performed in LipidSearch software 4.2. Metaboanalyst 4.0 was used for statistical analysis (Fig. 3 and Fig. 4).

## 2 Experimental Design, Materials and Methods

### 2.1 Animals

All surgical procedures were performed in compliance with animal protocols approved by the IACUC at Boston Children's Hospital. Mice were anaesthetized with ketamine and xylazine and received buprenorphine as a postoperative analgesic. For AAV injection, 4-week-old Pten^loxP/loxP^ mice were intravitreally-injected with 2–3 μl of either AAV-Cre (KO) or AAV-PLAP (control) with a pulled glass micropipette attached to a Hamilton syringe (Hamilton, Reno, NV). For intravitreal injections, the pulled-glass micropipette was inserted near the peripheral retina behind the ora serrata and deliberately angled to avoid damage to the lens. Optic nerve crush (ONC) injury was performed two weeks after AAV injection, as per previously described[1]. Briefly, the optic nerve was exposed at the intra orbital space and crushed with fine forceps (Dumont #5, Fine Science Tools, Foster City, CA) for 5 s approximately 500 mm behind the optic disc. Eye ointment was applied post-operatively to protect the cornea. At indicated time-points, mice were euthanized, and optic nerves were immediately dissected out, and then flash frozen on dry ice.

### 2.2 Optic nerve regeneration visualization

For anterograde labeling of retinal ganglion cell axons within the optic nerve, 1 μl of cholera toxin subunit B with a conjugated Alexfluor555 (1 µg/µl in sterile saline, Thermo Fisher Scientific, Waltham, MA) was injected intravitreally two days before perfusion. Dissected optic nerves were cleared using Visikol clearing solutions (per manufacturer's instructions). Cleared optic nerves anterogradely-labelled with CTB were then imaged using a Zeiss LSM 710 multiphoton confocal microscope.

### 2.3 Lipid extraction

Lipids were isolated from optic nerves subjected to an alternating cycle of freeze at -80°C and thaw at 37°C, followed by the Bligh and Dyer [2] method. The lower organic phase containing the lipids was removed and dried in a Speed-Vac (Model 7810014; Labconco, Kansas City, MO). To prevent lipid oxidation, the tubes were flushed with argon gas for storage. The upper phase was used to measure the sample protein concentration with a BCA Protein Assay Kit (ab102536).

### 2.4 High performance liquid chromatography and mass spectrometry

The dried lipid samples were resuspended in 50 µl of chloroform: methanol 1:1 (v/v), placed in an ultrasonic water bath for 20 minutes. From each sample, 24 µl were added to two vials, of which one contained 2 ug/ml of EquiSPLASH™ LIPIDOMIX® Quantitative Mass Spec Internal Standard (330731). An Accela Autosampler and Accela 600 pump (Thermo) was used for reverse phase separation with an Acclaim column (120 C18 3 µm). The Q Exactive (Thermo) mass spectrometer was operated under heated electrospray ionization (HESI) and samples were ran in both positive and negative mode separately. The solvents were LC-MS grade methanol: water 60:40 (v/v) with 10 mM ammonium acetate and methanol chloroform 60:40 (v/v) with 10 mM ammonium acetate.

### 2.5 Lipid identification and statistical analysis

The *.RAW scans were analysed with LipidSearch 4.2 software (Thermo). The parameters were set to M-score 2 and product search. The quantification was carried by the internal standard classes identified and protein concentration from upper phase of extraction. In Metaboanalyst 4.0, the autoscale feature was employed (mean-centered and divided by standard deviation of each variable).

## Ethics Statement

Studies in humans and animals. This study utilized animals (mouse, encompassing both genders) only. All animal experiments were performed in compliance with the US National Institutes of Health guide for the care and use of Laboratory animals. The sex of the mouse is not known to influence or have an association with optic nerve neuron regeneration.

## CRediT Author Statement

Jennifer Arcuri: Experimentation, Data curation, Writing– Original draft preparation; Shane Hegarty: Animal experimentation, Writing–Reviewing and Editing; Zhigang He: Resource, Methodology, Supervision, Writing–Reviewing and Editing; Sanjoy K. Bhattacharya: Conceptualization, Methodology, Software, Writing– Reviewing and Editing.

## Declaration of Competing Interest

The authors declare that they have no known competing financial interests or personal relationships which have, or could be perceived to have, influenced the work reported in this article.

## Appendix Supplementary materials

Image, application 1

## References

[1] Park K.K. (2008). Promoting axon regeneration in the adult CNS by modulation of the PTEN/mTOR pathway. Science.

[2] Bligh E.G., Dyer W.J. (1959). A rapid method of total lipid extraction and purification. Can J. Biochem. Physiol..

[3] Trzeciecka A. (2019). Comparative lipid profiling dataset of the inflammation-induced optic nerve regeneration. Data Brief.

[4] Trzeciecka A. (2019). Lipid profiling dataset of the Wnt3a-induced optic nerve regeneration. Data Brief..

[5] Arcuri J. (2020). Lipid profile dataset of optogenetics induced optic nerve regeneration. Data Brief..

